# A base measure of precision for protein stability predictors: structural sensitivity

**DOI:** 10.1186/s12859-021-04030-w

**Published:** 2021-02-25

**Authors:** Octav Caldararu, Tom L. Blundell, Kasper P. Kepp

**Affiliations:** 1 DTU Chemistry, Technical University of Denmark, Building 206, 2800 Kgs. Lyngby, Denmark; 2 Department of Biochemistry, University of Cambridge, Cambridge, CB2 1GA UK

**Keywords:** Protein stability, Mutation, Computation, Protein structure, Structural sensitivity

## Abstract

**Background:**

Prediction of the change in fold stability (ΔΔG) of a protein upon mutation is of major importance to protein engineering and screening of disease-causing variants. Many prediction methods can use 3D structural information to predict ΔΔG. While the performance of these methods has been extensively studied, a new problem has arisen due to the abundance of crystal structures: How precise are these methods in terms of structure input used, which structure should be used, and how much does it matter? Thus, there is a need to quantify the structural sensitivity of protein stability prediction methods.

**Results:**

We computed the structural sensitivity of six widely-used prediction methods by use of saturated computational mutagenesis on a diverse set of 87 structures of 25 proteins. Our results show that structural sensitivity varies massively and surprisingly falls into two very distinct groups, with methods that take detailed account of the local environment showing a sensitivity of ~ 0.6 to 0.8 kcal/mol, whereas machine-learning methods display much lower sensitivity (~ 0.1 kcal/mol). We also observe that the precision correlates with the accuracy for mutation-type-balanced data sets but not generally reported accuracy of the methods, indicating the importance of mutation-type balance in both contexts.

**Conclusions:**

The structural sensitivity of stability prediction methods varies greatly and is caused mainly by the models and less by the actual protein structural differences. As a new recommended standard, we therefore suggest that ΔΔG values are evaluated on three protein structures when available and the associated standard deviation reported, to emphasize not just the accuracy but also the precision of the method in a specific study. Our observation that machine-learning methods deemphasize structure may indicate that folded wild-type structures alone, without the folded mutant and unfolded structures, only add modest value for assessing protein stability effects, and that side-chain-sensitive methods overstate the significance of the folded wild-type structure.

## Background

The accurate prediction of the change in protein fold stability (ΔΔG) upon amino-acid substitution is a central challenge in modern biology, the solution to which would enable efficient rational engineering of stable proteins for industry and medicine [1–3], help us to understand protein evolution where stability effects play a major role [4–9], and improve our understanding of many protein-stability related genetic diseases driven by point mutations [10–12].

Many programs can predict ΔΔG by utilizing diverse prediction models from machine-learning to energy-based force-fields [13–22]. An important distinction can be made between those methods that only use the protein amino-acid sequence to predict stability and those that use a three-dimensional wild-type structure as input. Logically, one expects methods that use a 3D structure to perform better, since interactions between amino acids cannot be fully deduced from sequence alone. However, the structure-based methods importantly miss both the unfolded wild-type structure, and the folded and unfolded mutants structures, i.e. three of the four structures of the thermodynamic cycle of ΔΔG values; perhaps partly for this reason, structure-based methods perform only slightly better than their sequence-based counterparts [23].

The worse-than-expected performance of structure-based methods can also relate directly to the quality of the structures used. Indeed, it has been long debated whether crystal structures reproduce the native structures of proteins in solution and cells, as structures could be affected by crystal packing effects [24, 25]. Thus, while we can say whether a structure is more precisely determined, it is difficult to say which protein structure is more realistic. Databases such as ProTherm [26] and VariBench [27] annotate each experimental data point with a Protein Data Bank (PDB) [28] code that may not represent the best structure if more structures are available, and this could affect the computed ΔΔG value.

It is well-known that energy-based methods such as FoldX [29] or Rosetta [30] can be quite affected by the structure used, and the authors of the methods recommend to minimize the structures used as input before prediction. On the other hand, machine learning methods, which arguably deemphasize the protein structure relative to such methods (as shown clearly below), have been previously indicated to be less structurally sensitive, at least for certain proteins [31, 32].

In addition to the accuracy (as estimated from the performance for a balanced data set) [33], it is therefore also important to know how sensitive a method is to the input structure used, i.e. its “precision”. Method accuracy has been extensively studied and the moderate accuracy of the prediction methods was attributed to failings in the scoring functions [17], inability to correctly predict stabilizing mutations [23] or biases originating from the datasets used for training [33]. An important set of principles for evaluating protein stability prediction methods of the prediction methods was reported by Niroula and Vihinen [34], but the impact of structure choice on the computed outcome was not discussed, yet, as shown below, adds an additional criteria for evaluating these methods. To our knowledge, structural sensitivity has only been considered when studying select proteins such as superoxide dismutase [31] and myoglobin [32].

A more generally confirmed structural sensitivity measure would represent a base measure for the precision of the methods, which seems required from a scientific point of view, since accuracy without precision does not suffice to establish predictive power, and any output should ideally be seen in the context of such a precision measure, i.e. the noise expected due to choice of structural input. However, it should be noted that a method showing no structural sensitivity at all is not desirable either, as it would imply that the method underemphasizes the wild type structure, leaving it practically if not formally a sequence-based method.

In this paper we studied the structural sensitivity of six widely used protein stability prediction methods, including both energy and knowledge-based methods and machine learning methods. The structural sensitivity was determined from computational saturated mutagenesis [35] applied on 25 different proteins, each having multiple possible structures published in the PDB. Our results show that structural sensitivity varies greatly, with some methods showing high sensitivity (~ 0.6 to 0.8 kcal/mol) whereas others, notably all studied machine-learning methods, are very insensitive to structure choice (~ 0.1 kcal/mol). Furthermore, this sensitivity was rather constant across the proteins studied, showing that the models themselves cause the behaviour regardless of the structural heterogeneity itself. Our results provide a good baseline for the precision of protein stability calculators useful for future studies. As a consequence, we recommend the use of triplicate ΔΔG evaluations on three distinct structures and reporting of the associated standard deviation whenever this is possible.

## Methods

### Protein structures studied

23 proteins were selected from an exhaustive search of PDB structures that fulfilled the following conditions:

- At least three crystal structures (3–5 used) in the PDB at different resolutions (at least 0.15 Å standard deviation of the resolution among the structures) but with the same amino-acid sequence;
- All structures monomeric in the crystal form;
- All structures apo (no ligands) in the crystal form;
- No missing CA atoms, except for N-terminal or C-terminal residues.

Furthermore, one protein with a metal centre (carbonic anhydrase) and one protein in a tetrameric form (deoxy-haemoglobin) were included to test the influence of such substantial features on structural sensitivity, for a total of 25 proteins. All structures were renumbered so that the same number corresponds to the same residue in all structures of a protein. More than three structures were selected for proteins with a wide spread of resolutions, to a maximum of five structures for Lyz, CAH, Rnase and UBQ. This resulted in a varied list of long and short proteins and proteins belonging to all secondary structure classes in CATH, which should make our results more general; however, importantly it turns out that these variations have modest effect, i.e. the sensitivities are quite generic to the methods. The full list of proteins and their PDB IDs is given in Table 1.

**Table 1:** List of proteins studied in this work

Name	Abbreviation	CATH	Length (*N*)	PDB IDs	Average resolution	STDEV resolution
Human acidic fibroblast growth factor	FGF	β	513	2AFG, 3UD7, 1JQZ, 1RG8	1.88	0.71
Barnase	Bar	α + β	110	1BNI, 1B2X, 1A2P, 1X1X	1.32	0.35
Hen Lyzozyme	Lyz	α	129	2LYZ, 2VB1, 3LZT, 5LIN, 5HNL	1.50	0.73
Beta Lactamase	BLac	α + β	263	1ZG4, 1BTL, 4OQG	1.31	0.44
Alpha Amylase	AAmy	α + β	453	1AQM, 1AQH, 1B0I	2.08	0.28
Ubiquitin	UBQ	α + β	76	1UBQ, 6Q00, 4XOK, 4XOL, 4XOF	1.78	0.83
Bovine pancreatic trypsin inhibitor	BPTI	NoSS	58	1BPI, 3LDJ, 9PTI	1.34	0.32
Carbonic anhydrase	CAH	α + β	258	1CAH, 3KS3, 6PEA, 1CA2, 1HCA	1.69	0.56
Hemoglobin	Hem	α	574	4HHB, 2DN2, 2HBS, 1COH	1.39	0.69
Ribonuclease A	Rnase	α + β	108	6ETK, 1KF4, 2BLP, 1C0B, 1JVV	1.43	0.56
Thaumatin	Thm	β	207	5AVG, 1THI, 1PP3	1.32	1.16
Human serum albumin	HSA	α	578	4G03, 1AO6, 4K2C	2.65	0.53
Turkey lysozyme	MLyz	α	129	2LZ2, 3LZ2, 135L	2.00	0.62
T-cell glycoprotein	TGly	β	363	1WIQ, 1WIP, 1WIO	4.3	0.61
Uracil-DNA Glycosylase	UGlc	α + β	223	2OYT, 2OXM, 1EMH, 1AKZ	1.37	0.40
Beta-secretase 1	BACE1	β	370	2ZHS, 2ZHT, 2ZHV	2.30	0.42
Toxic shock toxin 1	TST1	β	194	5TSS, 2TSS, 2QIL	2.34	0.48
Human Procathepsin	HPC	α + β	317	1PBH, 2PBH, 3PBH	3.01	0.44
Gamma-B Crystallin	GBC	β	174	4GCR, 1GCS, 1AMM	1.56	0.41
Thermomyces Lanuginosa Lipase	TLL	α + β	269	1TIB, 1DTE, 1DT3	2.26	0.39
Spasmolytic polypeptide	SPP	NoSS	105	1POS, 1PSP, 2PSP	1.35	0.35
Human neonatal Fc receptor	FCR	β	263	3M17, 3M1B, 5BJT	2.37	0.32
Cyclophilin A	CycA	β	164	4YUJ, 5F66, 5KUL	1.42	0.28
Lysin	Lys	α	131	1LIS, 2LIS, 5II9	1.79	0.39
Tryparedoxin I	TRR	α + β	143	1EWX, 1O7U, 1QK8	1.53	0.16

CATH secondary structure class, PDB IDs, and the average and standard deviation among all the resolution values

### Structural sensitivity calculations

In order to calculate the structural sensitivity, a saturated computational mutagenesis [35] (all amino-acids were mutated to all other 19 amino-acids to produce 19* N* mutants) was performed starting from all PDB structures with all six prediction methods, with a total of 87 structures subjected to saturated computational mutagenesis.

Structural sensitivity per mutation (*SS*_*mut*_) was defined as the standard deviation of the predicted ΔΔG values (in kcal/mol) for one mutation among all PDB structures of the same protein. Structural sensitivity per site (*SS*_*site*_) was defined as the average *SS*_*mut*_ (in kcal/mol) for all mutations from a specific residue of one protein, i.e. the average of 19 mutations. Structural sensitivity per protein (*SS*_*prot*_) was defined as the average *SS*_*mut*_ (kcal/mol) for all mutations in a specific protein.

### Prediction methods studied

The studied prediction methods were selected based on their ability to model any mutation, to give a quantitative ΔΔG prediction (rather than just qualitatively; destabilizing or stabilizing) and to work at high computational speed so that saturated computational mutagenesis was feasible. Structural sensitivity should depend on the model used for ΔΔG prediction, and accordingly a diverse group of methods was desired to assess sensitivity broadly. Six publicly available predictors were explored in this study: FoldX [36], I-Mutant 3.0 [37], PoPMuSiC 2.1 [38], Maestro [39], mCSM [19] and CUPSAT [40].

The chosen methods use a variety of algorithms to compute the change in protein stability upon mutation: FoldX uses an empirical force field to calculate the free energy of folding for the wild-type and mutant structures. As the force field is rather sensitive to structure [41], a minimization of the wild-type structure was performed before prediction using the FoldX command *RepairPDB*. The differences between the minimized structures from *RepairPDB* and the original PDB structures are very small, with the maximum all-atom root mean square deviation (RMSD) < 0.01 Å (Additional file 1: Table S1), thus FoldX’s structural sensitivity can be compared with the other methods. CUPSAT uses atomic potentials from chemical properties and empirically derived torsion potentials. I-Mutant 3.0 uses support-vector machines that account for amino acid substitution and structural environments. Similarly, Maestro combines support vector machines with a random-forest approach to obtain a consensus free energy. mCSM uses graph-based signatures that encode distance patterns between atoms. PoPMuSiC uses a statistical potential calculated from contact probabilities of amino acids close to the mutated residue. Thus, we studied one energy-based method (FoldX), two knowledge-based methods (PoPMuSiC and CUPSAT) and three machine learning methods (mCSM, I-Mutant 3.0 and Maestro).

Unless specified otherwise, all prediction programs were run with default parameters.

### Calculating global and local structural variables of amino-acids

The secondary-structure composition of each protein was taken from CATH [42]. The length of the proteins (*N*) was considered as the number of amino-acids in the structure. Pairwise root-mean-square deviation (RMSD) was calculated with the PyMol [43] command *rms_cur* after alignment of the structures. The average RMSD is reported as the average of all pairwise RMSD values for one protein.

The secondary structure of each amino-acid was calculated using the *dssp* program [44] and then converted to a four-category secondary structure. The secondary structure per residue is reported as the consensus *dssp* calculation for all PDB structures used. The RMSD per residues is the average pairwise RMSD per residue between all structures of the same protein, calculated with the PyMol script RmsdByResidue after alignment. Relative solvent accessibility (RSA) was calculated with Naccess [45, 46], using default van der Waals atomic radii, and is reported as the average accessibility for all PDB structures of the same protein. Cystine bridges in each structure were predicted using the DisulfideByDesign 2.0 server [47, 48].

## Results

### Structural sensitivity measured for the full proteins

**Fig. 1:**
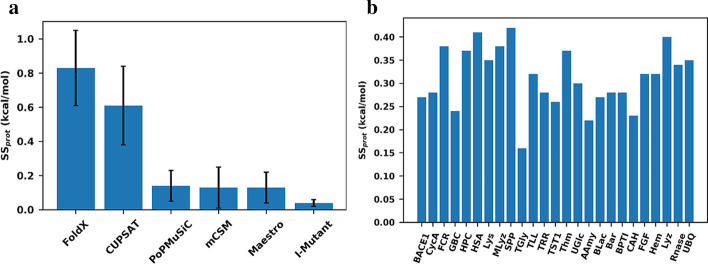
Structural sensitivity. **a** Average structural sensitivity per protein for each prediction method. Error bars show standard deviation of structural sensitivity among all proteins. **b** Average structural sensitivity of all prediction methods for each protein studied

The 25 proteins were subjected to computational saturated mutagenesis, started from each of the selected structures for each protein. Figure 1a shows the average *SS*_*prot*_ for all six methods and the standard deviation. The individual data for each protein can also be found in Additional file 1: Table S1. Of the six methods, CUPSAT and FoldX displayed much larger structural sensitivity than the other four methods, i.e. *SS*_*prot*_ = 0.83 kcal/mol and 0.61 kcal/mol, respectively. FoldX is an energy-based method that applies many terms in its energy function, such as electrostatic and van der Waals forces, whereas CUPSAT is a knowledge-based method that bases its energy calculation on statistics of torsional angles, which are very sensitive to differences in side-chain structure. We conclude that the magnitude of the structural sensitivity for these two methods is quite worrying, since, in perspective, the average ΔΔG for any typical, random mutation is perhaps + 1.0 kcal/mol on average. Thus, CUPSAT and FoldX come with an intrinsic imprecision that approaches the actual predicted value. We note that this does not necessarily imply lower trend accuracy, but it will certainly affect the predictive capacities of the methods. CUPSAT and FoldX also displayed the largest differences in structural sensitivity across the protein structures used, with a standard deviation of more than 0.2 kcal/mol in total for the 25 studied proteins.

In contrast, the other four studied methods displayed quite modest structural sensitivity, ranging from 0.04 kcal/mol for I-Mutant 3.0 to 0.14 kcal/mol for PoPMuSiC 2.0. I-Mutant, mCSM and Maestro are all machine-learning methods that do not take into account detailed features of the local environment, whereas PoPMuSiC is a knowledge-based method that captures interactions between close and distant amino-acids, but it is heavily parametrized, which can perhaps explain the much lower structural sensitivity compared to CUPSAT. Interestingly, PoPMuSiC, mCSM and Maestro all showed almost the same average structural sensitivity. Conversely, I-Mutant was very structurally insensitive, and produced very little differences between each protein. Although this means that any 3D structure can be used for prediction with I-Mutant, it also suggests that I-Mutant underreports structural information, i.e. is practically very close to a sequence-based method. Our analysis thus inspires a more quantitative view on sequence-versus structure-based methods than just qualitative yes/no, with methods lying on a spectrum, as seen from their actual structure sensitivity.

Figure 1b shows the average *SS*_*prot*_ for each protein among all six methods. Surprisingly, the structural sensitivity did not vary much between proteins, with all showing averages around 0.3 ± 0.1 kcal/mol, except one case, TGly, which has the lowest average resolution of the structures used (4.3 Å), indicating that structural sensitivity is low when all structures are of poor quality. This low variance is confirmed by the low standard deviation of the structural sensitivity of the methods (Fig. 1a and Additional file 1: Table S1) and by a single-factor analysis of variance (ANOVA) performed for all 25 protein (Additional file 1: Table S3). This tendency is observed regardless of the variable quality of the structures used, regardless of the different spreads in resolution for each protein, and largely regardless of the presence of metal sites, as for Hem and CAH. Furthermore, the proteins for which more than three structures were used did not exhibit distinct structural sensitivities. These results indicate that structural sensitivity depends mostly on the prediction model itself and not so much on the features and differences between the protein structures selected for our study.

**Fig. 2:**
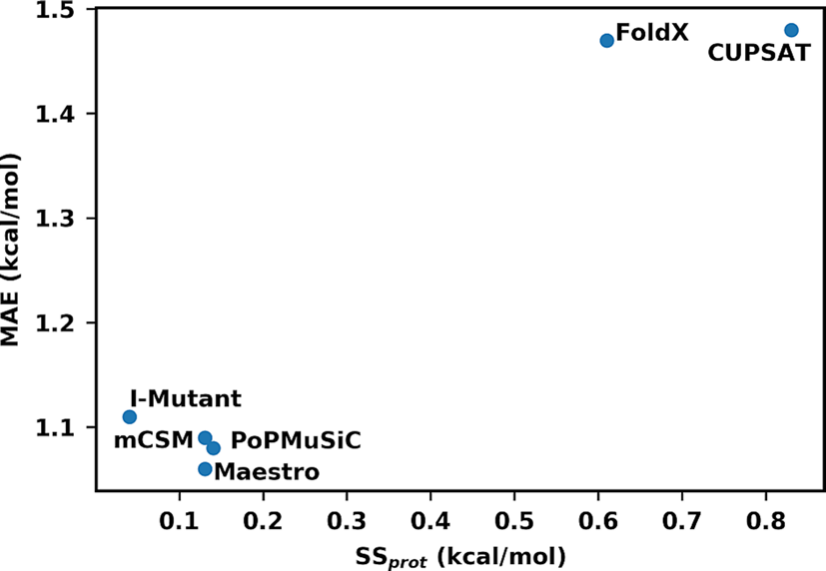
Accuracy and precision of studied methods. Mean absolute deviation (MAE, in kcal/mol) of the six methods against the balanced O2567 data set, as calculated in Caldararu et al*.* [33] versus average structural sensitivity per protein (in kcal/mol) for each prediction method

Comparing the computed precision of the six methods with the accuracy (mean absolute error) calculated in our previous study for a mutation-type balanced data set [33] reveals that the two methods with higher structural sensitivity also display lower accuracy for balanced data (Fig. 2). This suggests that accuracy and precision are correlated, and that structural sensitivity may play a part in the accuracy of the methods, along with the data set bias previously identified. It is therefore important to carefully select the protein structures used for training the models and assess the impact of structural sensitivity during training. We note that CUPSAT and FoldX still work quite well for trend predictions, as they retain accuracy and precision locally for certain mutations, as shown in previous benchmarks.

### Factors that affect structural sensitivity

In order to understand and control the structural sensitivity issue identified in Fig. 1, we must first understand the factors that influence the structural sensitivity of each method, for example whether certain proteins are more structurally sensitive than others. Moreover, mutation studies often focus on certain sites and residues in a protein, and thus it is important to determine which type of residues are mostly contributing structural sensitivity during prediction.

**Fig. 3:**
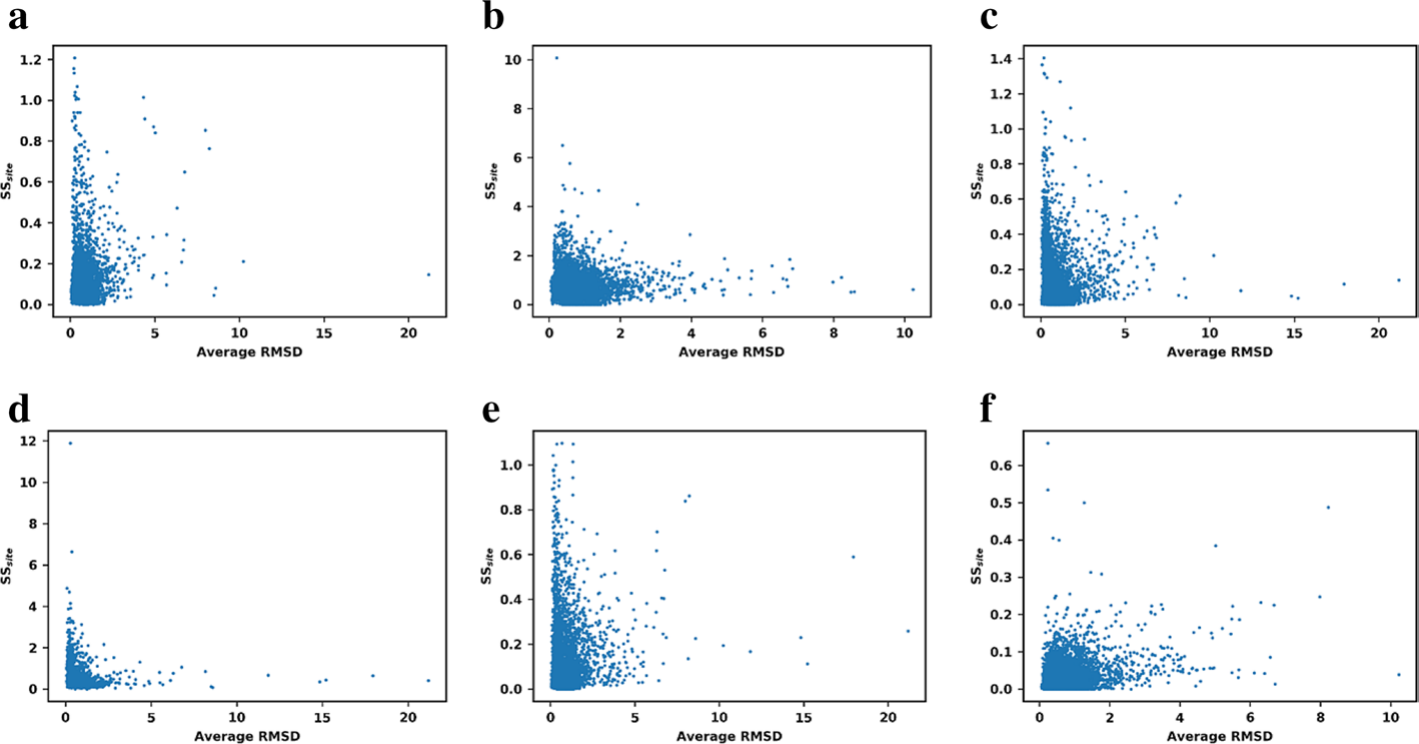
Relation between structural sensitivity and RMSD. Structural sensitivity per site (in kcal/mol) versus Average RMSD per residue among all residues in all 25 proteins for: **a** PoPMuSiC; **b** CUPSAT; **c** mCSM; **d** FoldX; **e** Maestro, **f** I-Mutant

The most logical cause of structural sensitivity is the structural difference between the PDB structures used, which can be measured by the average RMSD between all structures. To determine whether amino-acids that are in different conformations in different structures display larger differences than amino-acids in the same conformation we plotted *SS*_*site*_ for all the residues in all 25 proteins against the average RMSD per residue (Fig. 3). Strikingly, no methods showed any correlation between RMSD and structural sensitivity. Actually, most sites that had high structural sensitivity also had an RMSD relatively close to 0, i.e. the conformation of the residues was the same in all structures used. Furthermore, rigid residues (residues with a B-factor close to the average B-factor of the protein) were also found to have higher structural sensitivity than flexible residues (Additional file 1: Fig. S1**)**. Although this might at first seem counter-intuitive, it implies that prediction methods are more structurally sensitive to buried residues, which usually have the same conformation in all structures of the same protein and are more rigid, and usually associated with larger energy effects. More flexible residues on the other hand typically reflect low-energy modes such as rotations, which may not affect ΔΔG as much.

**Fig. 4:**
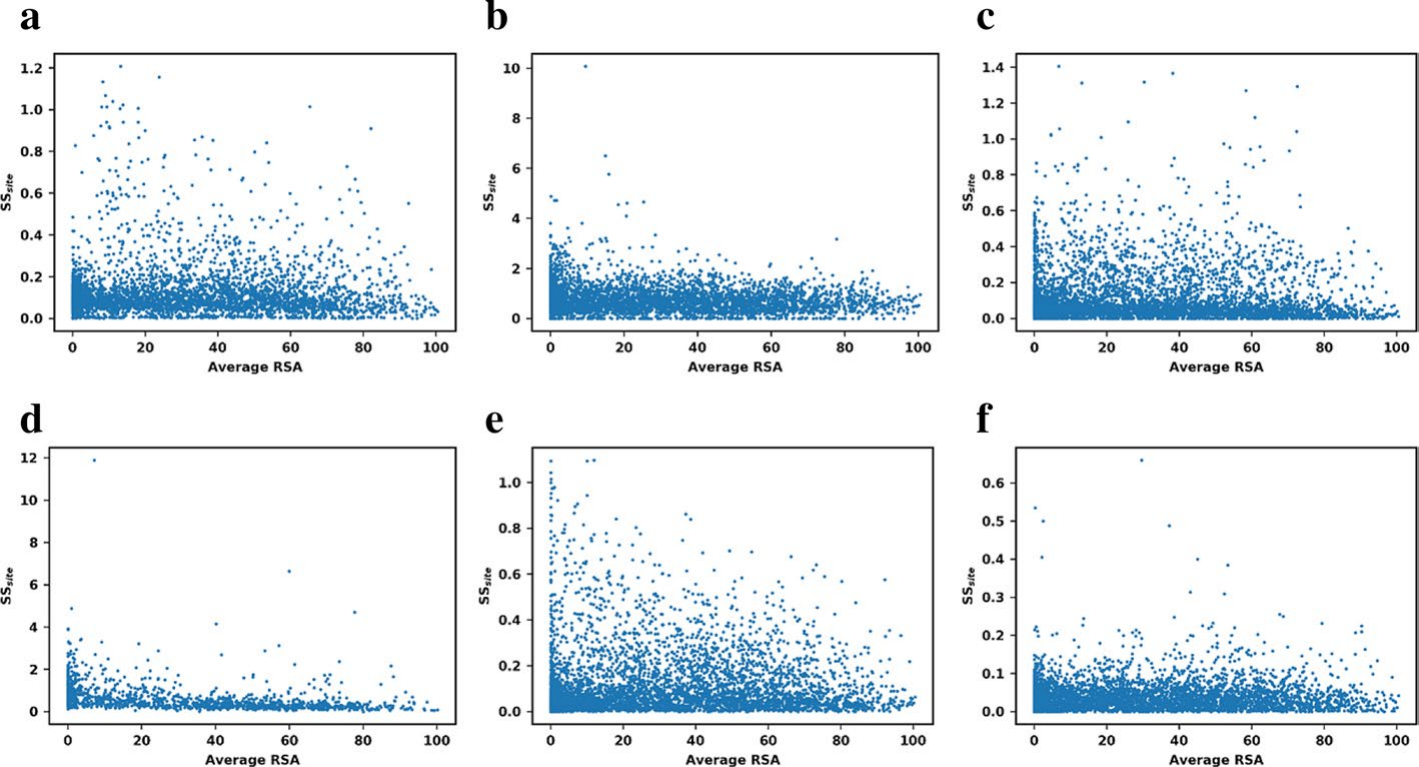
Structural sensitivity per site (in kcal/mol) versus Average RSA per residue. The plots show all residues in all 25 proteins for the methods: **a** PoPMuSiC; **b** CUPSAT; **c** mCSM; **d** FoldX; **e** Maestro, **f** I-Mutant

For these reasons we also studied the relationship between the relative solvent accessibility (RSA) of each residue and *SS*_*site*_. The results, illustrated in Fig. 4, again revealed no correlation between the RSA and the structural sensitivity for any of the methods. Several outliers for FoldX and I-Mutant are indeed buried residues, but we observed no general trend.

**Fig. 5:**
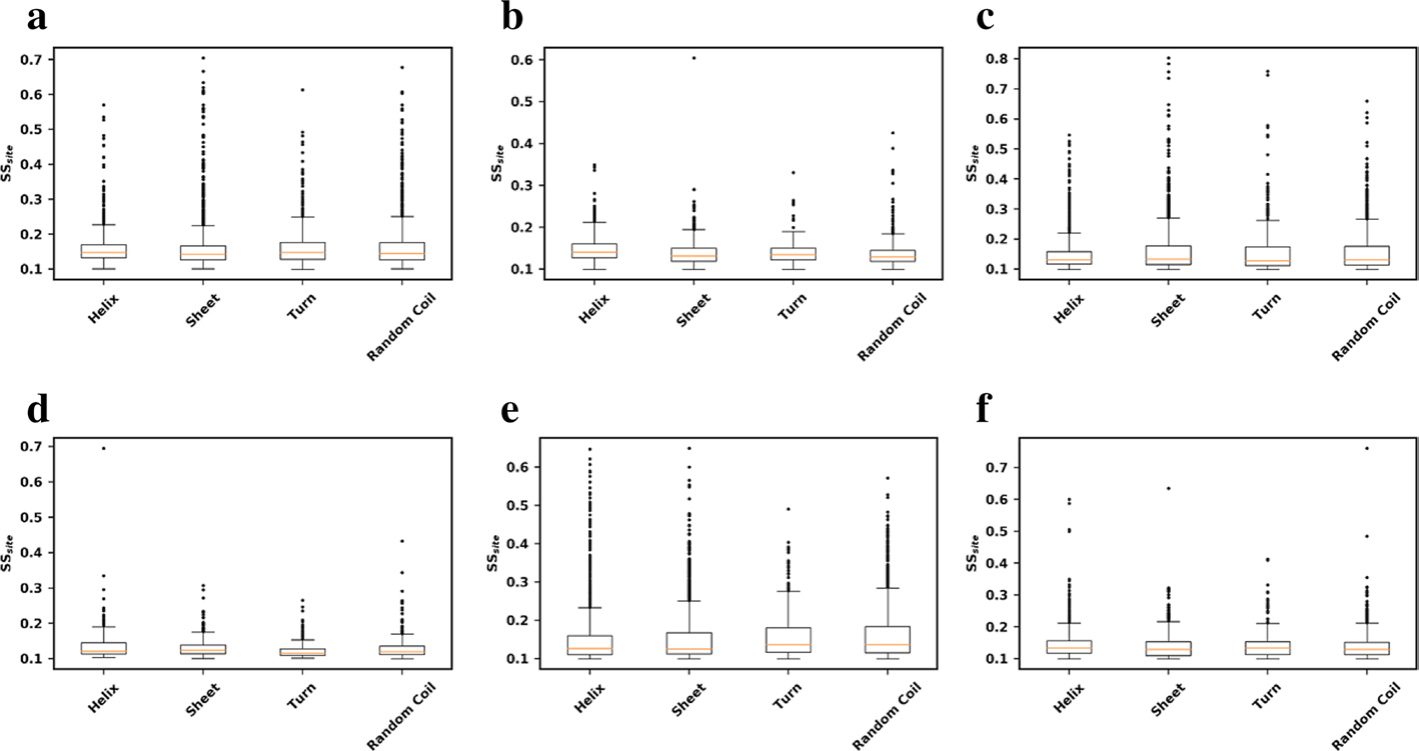
Structural sensitivity per site (in kcal/mol) for residues in different secondary structures. α-helix, β-sheet, turn or random coil among all residues in all 25 proteins for: **a** PoPMuSiC; **b** CUPSAT; **c** mCSM; **d** FoldX; **e** Maestro, **f** I-Mutant

Next, we investigated whether the secondary structure of each residue influenced structural sensitivity, as many prediction methods contain a term for the secondary structure of the wild-type residue during ΔΔG calculation. As shown in Fig. 5, the average *SS*_*site*_ for all residues in the 25 proteins was similar for all four types of secondary structure in all six prediction methods, with outliers also being similarly shared across the four types of secondary structure.

In a previous study, we showed that mutation type greatly affects method accuracy, and none of the studied methods were generally transferable and balanced in this context [33]. Therefore, we studied in the present work whether mutation type also affects the precision (i.e. structural sensitivity) of the methods. A mutation type was considered to have high sensitivity if its average *SS*_*mut*_ was significantly higher than the average for the method, as shown in Fig. 1.

**Fig. 6:**
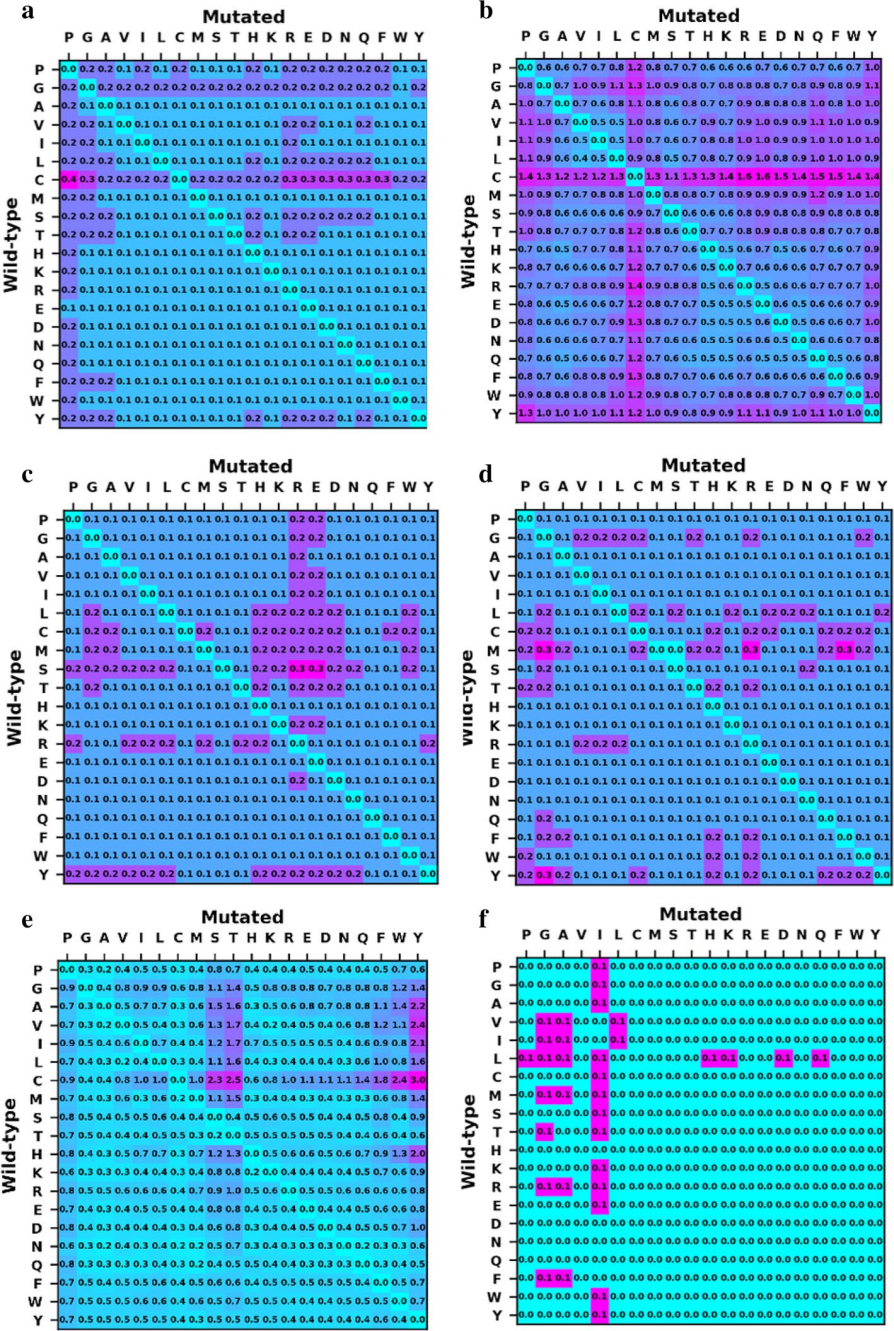
Structural sensitivity per mutation. Values shown in kcal/mol for all mutations of a certain mutation type among all mutations studied in all 25 proteins for: **a** PoPMuSiC; **b** CUPSAT; **c** mCSM; **d** FoldX; **e** Maestro, **f** I-Mutant

Figure 6 shows the average *SS*_*mut*_ for each of the 380 mutation types in all 25 proteins and for each of the prediction methods. We observe that each prediction method is more structurally sensitive for certain mutation types. PoPMuSiC is sensitive to mutations from C (Fig. 6a), whereas CUPSAT has higher sensitivity for all mutations involving C (Fig. 6b). mCSM presented higher sensitivity for mutations involving charged residues, especially R and E (Fig. 6c). FoldX showed the most substantial differences, with mutations to hydroxyl-containing amino-acids (S, T, Y) having significantly higher structural sensitivity than other mutations (Fig. 6d). Maestro (Fig. 6e) and I-Mutant (Fig. 6f) were more balanced, although Maestro had a slightly higher structural sensitivity for mutations from M and I-Mutant showed a similar behaviour for mutations to I.

Although none of the structures used in this study had any explicit cystine bridges in their PDB files, we investigated more closely if cystine bridges could occur in the selected proteins, since two methods showed particularly high structural sensitivity for mutations from C. We separated the data into two separate data sets, proteins with predicted cystine bridges and proteins without any predicted cystine bridges (Additional file 1: Table S4) and recalculated the average *SS*_*mut*_ for each mutation type for CUPSAT and PoPMuSiC (Additional file 1: Fig. S2). Our results indicate that the sensitivity of PoPMuSiC towards mutations from C may be caused by possible cystine bridges, whereas CUPSAT was not affected by the presence of cystine bridges.

In summary, we conclude that among the properties potentially contributing to structural sensitivity studied here, the mutation type has the highest effect, and thus the precision of a method may be quite dependent on the mutation type studied, as we saw previously in terms of accuracy.

As a final note, we also studied several global parameters of the proteins: CATH structure (Additional file 1: Table S5), length and average global RMSD (Additional file 1: Table S6) but none showed any significant correlation to structural sensitivity. As *SS*_*site*_ has shown more variation than *SS*_*protein*_ it is expected that these global parameters will have little effect on the precision of the methods.

## Discussion

In this study, we have tried to evaluate the precision of commonly used protein stability prediction methods, defined as their structural sensitivity evaluated for all possible mutations in 25 proteins. Our results show that structural sensitivity varies substantially among the six studied methods and intriguingly cluster in two groups—those that are highly trained and those that are highly dependent on local environment of the mutation (FoldX and CUPSAT), which displayed high structural sensitivity (0.6 and 0.8 kcal/mol, respectively). This is as matter of concern since the average ΔΔG of a typical random mutation is of the order of ~ 1.0 kcal/mol. The methods still work to some extent because they retain accuracy locally in any structure used, but they are not very precise in our definition. On the other hand, machine-learning methods (mCSM, Maestro and I-Mutant) and parametrized knowledge-based methods (PoPMuSiC) are very insensitive to structure choice.

To deepen our understanding of structural sensitivity, we also studied what factors cause it. Our results indicate that there is no correlation between the structural sensitivity of residues and the RMSD between the structures used, probably because low-energy modes define much of the structural RMSD. Furthermore, neither the solvent accessibility, secondary structure nor B-factor values of the amino-acids had any significant correlation with structural sensitivity. Instead, the only factor that seems to affect precision was the mutation type, with different methods being more sensitive to different mutation types. Thus, structural sensitivity is caused by either the modelling of the wild-type structure or the parametrization bias towards some mutation types as touched upon in our recent related work [33]. The correlation in Fig. 2 shows that mutation-type is the most salient feature of both accuracy and precision and that none of these should be evaluated on mutation-type biased data if wider conclusions on performance are to be drawn. More importantly, and perhaps controversially, it is very interesting that all the studied machine-learning methods tend to train away almost entirely the used wild-type structure.

The least structure-sensitive methods are also the most accurate for our balanced benchmark data, and their structural sensitivity (~ 0.1 kcal/mol) is within the experimental uncertainty in the data. This may controversially indicate that folded wild type structures provide modest value to ΔΔG predictions, which, if true, could be because the three “other” structures of the thermodynamical cycle are missing. Some methods seem to overemphasize structure, probably side chain conformations, which may differ substantially in mutant and wild type unfolded states that produce the experimental data. We invite further studies to settle this question and also note that there is no “optimal” structural sensitivity except that which provides highest accuracy upon independent benchmarking.

## Conclusions

Users of stability prediction methods are often faced with a choice between many possible structures for a wild type protein of interest, and we wanted to explore in this study how this affects outcome. Our study provides a base measure of the precision of methods in relation to structure input used for calculations. This will be important both as a general tendency and for specific mutation types of distinct sensitivity in most studies using these methods, and thus we recommend that triplicate structures are used as input and the standard deviation of the ΔΔG reported as a best-practice for these methods, of course only if several reasonable structures are available. The specific structural sensitivities for each method and mutation type reveal the aspects needed to be improved in each method in order to optimize structural sensitivity.

Finally, instead of just considering sequence- and structure-based methods as either/or, our base measure provides a spectrum of actual structural emphasis of the methods, noting that a machine-learning could in principle “train away” structure completely even if used formally. The fact that all studied machine-learning methods are rather insensitive to structure input raises a perhaps controversial question on the relevance of folded wild-type structures alone (without folded mutant and unfolded structures) to ΔΔG prediction altogether. However, we show that a substantial reason for imprecision is, as for accuracy [33], bias from certain mutation types arising from training on imbalanced data sets, via their effect on local residue geometry. We do not claim to answer these questions in any completeness but given its importance suggest that this is explored further in future studies using other data sets.

## Supplementary Information

**Additional file 1.** The supporting information file (SuppInfo.pdf) contains full data for structural sensitivity per protein, and additional information on relations between structural sensitivity and B-factors and mutation types.

## Data Availability

The 3D protein structures that have been used in this study are publicly available online in the PDB, www.rcsb.org. The prediction programs used are freely available as servers or stand-alone programs. The website of each prediction program is listed below. FoldX: http://foldxsuite.crg.eu/. Maestro: https://pbwww.che.sbg.ac.at/maestro/web/. PoPMuSiC v 2.1: https://soft.dezyme.com/. CUPSAT: http://cupsat.tu-bs.de/. mCSM: http://biosig.unimelb.edu.au/mcsm/. I-Mutant: http://gpcr2.biocomp.unibo.it/cgi/predictors/I-Mutant3.0/I-Mutant3.0.cgi. Raw prediction data are available from the corresponding author upon request.

## Abbreviations

PDB: Protein Data Bank
SS_mut_: Structural sensitivity per mutation
SS_site_: Structural sensitivity per wild-type residue
SS_prot_: Structural sensitivity per protein
RMSD: Root mean square deviation
RSA: Relative solvent accessibility
