# Supplementary material for: A base measure of precision for protein stability predictors: structural sensitivity
Source: BMC Bioinformatics. 2021 Feb 25;22:88. doi: 10.1186/s12859-021-04030-w (PMC7908712; doi:10.1186/s12859-021-04030-w)
Supplement: Supplementary file 1 — Additional file 1. The supporting information file (SuppInfo.pdf) contains full data for structural sensitivity per protein, and additional information on relations between structural sensitivity and B-factors and mutation types. [file 12859_2021_4030_MOESM1_ESM.pdf]

Supplementary information

A Base Measure of Precision for Protein Stability Predictors: Structural Sensitivity

Octav Caldararu [1], Tom L. Blundell[2], and Kasper P. Kepp[1]*

[1] *DTU Chemistry, Technical University of Denmark, Building 206, 2800 Kgs. Lyngby, Denmark.*

[2] *Department of Biochemistry, University of Cambridge, Cambridge, CB2 1GA, United Kingdom.*

Correspondence: E-mail: kpj@kemi.dtu.dk. Tel: + +45 45252409

Table S1. All-atom root mean square deviation (in Å) between the original structures from the PDB and structures minimized with FoldX's module RepairPDB for all proteins in the study.

	PDB1	PDB2	PDB3	PDB4	PDB5	Average
BACE1	0.002	0.003	0.000			0.002
CycA	0.000	0.000	0.000			0.000
FCR	0.000	0.003	0.003			0.002
GBC	0.003	0.009	0.003			0.005
HPC	0.007	0.010	0.004			0.007
HSA	0.003	0.003	0.003			0.003
Lys	0.003	0.003	0.008			0.005
Mlyz	0.001	0.011	0.004			0.005
SPP	0.000	0.003	0.002			0.002
Tgly	0.007	0.007	0.007			0.007
TLL	0.000	0.001	0.000			0.000
TRR	0.000	0.000	0.000			0.000
TST1	0.001	0.001	0.004			0.002
Thm	0.000	0.000	0.000			0.000
Uglc	0.001	0.000	0.002	0.001		0.001
Aamy	0.000	0.000	0.000			0.000
Blac	0.000	0.000	0.001			0.000
Bar	0.000	0.001	0.004	0.003		0.002
BPTI	0.001	0.004	0.000			0.002
CAH	0.001	0.001	0.001	0.000	0.000	0.001
FGF	0.001	0.000	0.003	0.002		0.002
Hem	0.001	0.001	0.000	0.001		0.001
Lyz	0.007	0.000	0.000	0.001	0.002	0.002
Rnase	0.004	0.001	0.000	0.001	0.000	0.001
UBQ	0.002	0.000	0.002	0.001	0.000	0.001

Table S2. Structural sensitivity per protein (in kcal/mol) for each protein and each prediction method.

	PopMusic	CUPSAT	mCSM	FoldX	Maestro	I-Mutant	Average	STDEV
BACE1	0.11	0.67	0.06	0.57	0.19	0.04	0.27	0.27
CycA	0.08	0.59	0.42	0.48	0.09	0.04	0.28	0.24
FCR	0.14	1.07	0.10	0.80	0.14	0.06	0.38	0.44
GBC	0.08	0.65	0.10	0.48	0.07	0.03	0.24	0.26
HPC	0.18	1.13	0.10	0.66	0.14	0.03	0.37	0.43
HSA	0.09	1.00	0.06	1.24	0.03	0.04	0.41	0.56
Lys	0.11	0.83	0.13	0.59	0.42	0.03	0.35	0.32
Mlyz	0.16	1.00	0.07	0.85	0.15	0.05	0.38	0.43
SPP	0.39	0.73	0.31	0.78	0.29	0.03	0.42	0.29
Tgly	0.02	0.51	0.01	0.41	0.01	0.01	0.16	0.23
TLL	0.16	0.80	0.10	0.67	0.12	0.06	0.32	0.33
TRR	0.07	0.32	0.04	1.19	0.05	0.02	0.28	0.46
TST1	0.10	0.79	0.08	0.46	0.08	0.03	0.26	0.31
Thm	0.42	0.63	0.39	0.46	0.30	0.03	0.37	0.20
Uglc	0.13	0.91	0.08	0.52	0.12	0.05	0.30	0.35
Aamy	0.09	0.56	0.06	0.52	0.06	0.03	0.22	0.25
Blac	0.09	1.09	0.06	0.25	0.10	0.06	0.27	0.41
Bar	0.07	0.84	0.07	0.50	0.13	0.04	0.28	0.32
BPTI	0.14	0.87	0.09	0.45	0.07	0.06	0.28	0.32
CAH	0.11	0.68	0.09	0.41	0.08	0.04	0.23	0.26
FGF	0.13	1.13	0.01	0.48	0.11	0.03	0.32	0.43
Hem	0.11	1.10	0.05	0.52	0.08	0.04	0.32	0.43
Lyz	0.16	0.99	0.29	0.77	0.14	0.04	0.40	0.39
Rnase	0.16	1.00	0.18	0.50	0.12	0.07	0.34	0.36
UBQ	0.11	0.76	0.38	0.61	0.18	0.06	0.35	0.28
Average	0.14	0.83	0.13	0.61	0.13	0.04		
STDEV	0.09	0.22	0.12	0.23	0.09	0.02		

Table S3. ANOVA among structural sensitivities of all methods for each protein studied.

SUMMARY				
Groups	*Count*	*Sum*	*Average*	*Variance*
BACE1	6	1.92	0.27	0.06
CycA	6	1.98	0.28	0.05
FCR	6	2.69	0.38	0.16
GBC	6	1.65	0.24	0.06
HPC	6	2.61	0.37	0.16
HSA	6	2.87	0.41	0.26
Lys	6	2.47	0.35	0.08
Mlyz	6	2.66	0.38	0.15
SPP	6	2.96	0.42	0.07
Tgly	6	1.13	0.16	0.05
TLL	6	2.23	0.32	0.09
TRR	6	1.98	0.28	0.17
TST1	6	1.79	0.26	0.08
Thm	6	2.60	0.37	0.03
Uglc	6	2.10	0.30	0.10
Aamy	6	1.53	0.22	0.05
Blac	6	1.92	0.27	0.14
Bar	6	1.93	0.28	0.09
BPTI	6	1.97	0.28	0.09
CAH	6	1.64	0.23	0.05
FGF	6	2.21	0.32	0.16
Hem	6	2.21	0.32	0.15
Lyz	6	2.79	0.40	0.12
Rnase	6	2.36	0.34	0.11
UBQ	6	2.46	0.35	0.07

ANOVA						
Source of Variation	*SS*	*df*	*MS*	*F*	*P-value*	*F crit*
Between Groups	0.72	24	0.03	0.29	1.00	1.59
Within Groups	15.54	150	0.10			
Total	16.27	174				

Table S4. List of proteins containing predicted cysteine bridges (according to DisulfideByDesign 2.0) and proteins containing no predicted cystine bridges.

	Proteins
With Cystine Bridges	Lyz, BLac, AAmy, BPTI, Rnase, FCR, HPC, HSA, MLyz, SPP, TGly, TRR, Thm, TLL
No Cystine Bridges	FGF, Bar, UBQ, CAH, Hem, BACE1, CycA, GBC, Lys, TST1, UGlc

Table S5. Average structural sensitivity per protein for proteins belonging each CATH structure for the six prediction methods studied.

	PopMusic	CUPSAT	mCSM	FoldX	Maestro	I-Mutant
α	0.13	0.98	0.12	0.79	0.16	0.04
β	0.15	0.81	0.16	0.53	0.14	0.04
α+β	0.12	0.80	0.12	0.59	0.11	0.05
NoSS	0.46	1.17	0.36	1.01	0.33	0.06

Table S6. Pearson correlation coefficient of the structural sensitivity per protein for each method against various global parameters of the structures used.

	PopMusic	CUPSAT	mCSM	FoldX	Maestro	I-Mutant
Length	-0.22	0.24	-0.48	0.13	-0.17	0.01
RMSD	0.12	0.07	0.04	0.19	0.05	0.00

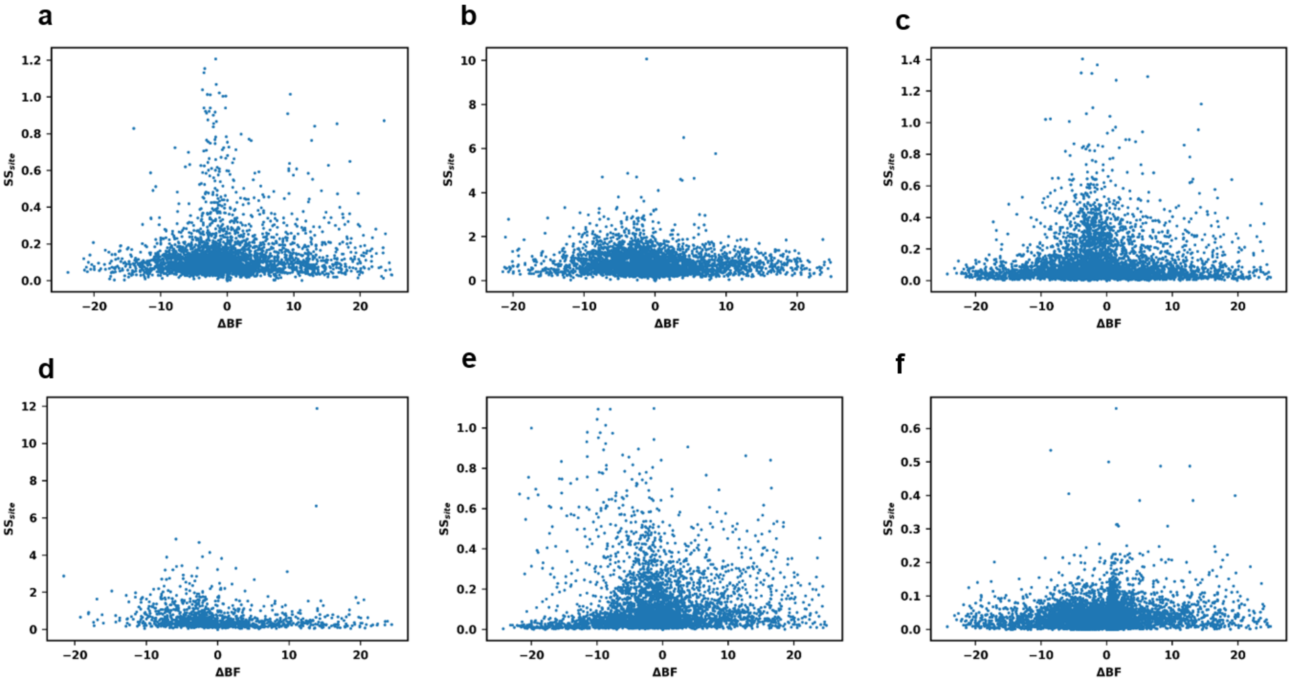

Figure S1. Structural sensitivity per site (in kcal/mol) vs ΔB-factor (the difference between the average residue B-factor and the average B-factor per protein) among all residues in all 25 proteins for: (a) PoPMuSiC; (b) CUPSAT; (c) mCSM; (d) FoldX; (e) Maestro and (f) I-Mutant.

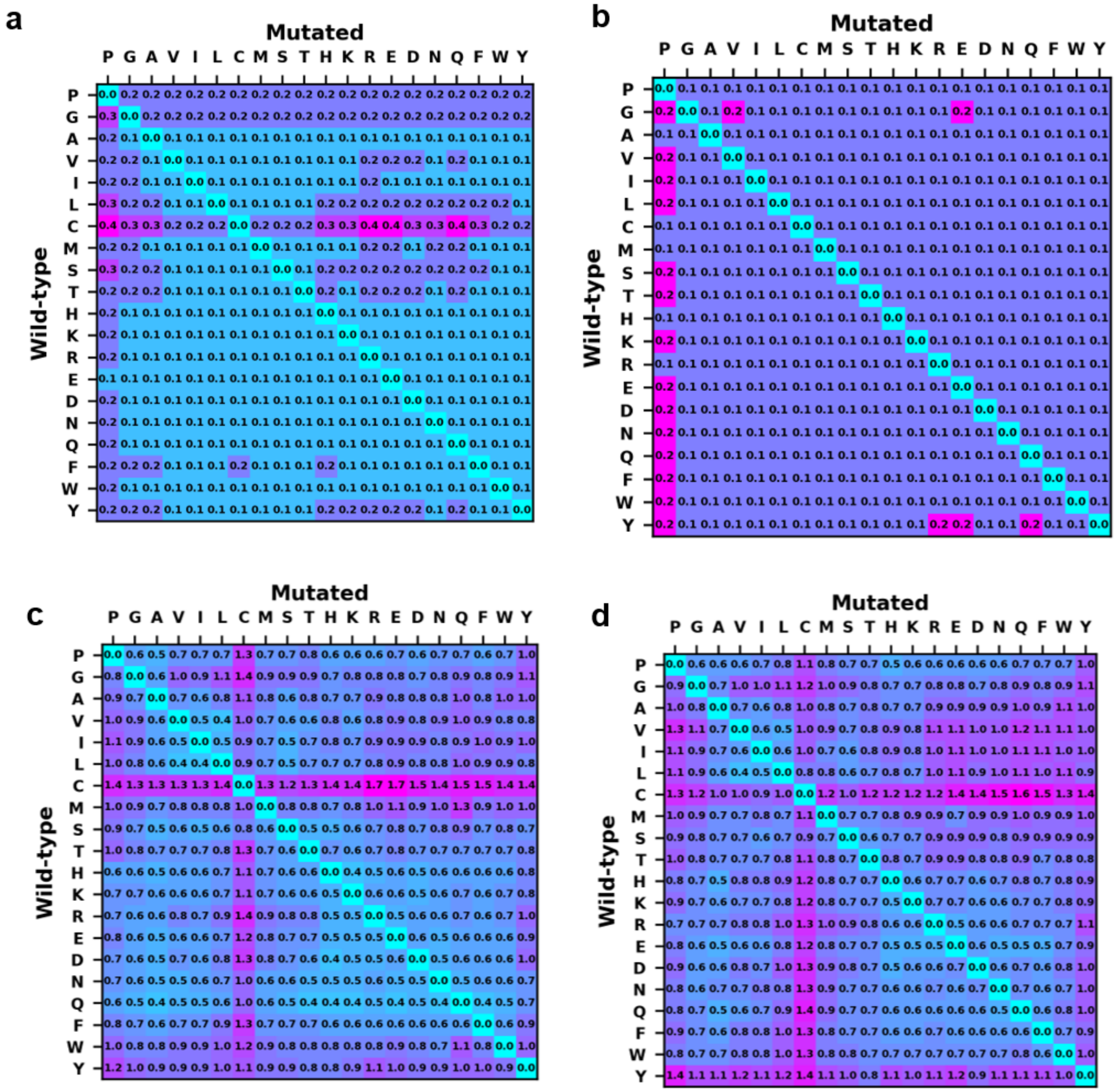

Figure S2. Structural sensitivity per mutation (in kcal/mol) for all mutations of a certain mutation type for (a) PoPMuSiC, on a dataset containing only proteins which contain predicted cystine bridges; (b) PoPMuSiC, on a dataset containing only proteins which do not contain any predicted cystine bridges; (c) CUPSAT; on a dataset containing only proteins which contain predicted cystine bridges; (d) CUPSAT; on a dataset containing only proteins which do not contain any predicted cystine bridges.
